# Dual-Planar Monopole Antenna-Based Remote Sensing System for Microwave Medical Applications

**DOI:** 10.3390/s24020328

**Published:** 2024-01-05

**Authors:** Minghui Zhao, Asad Riaz, Imran M. Saied, Zain Shami, Tughrul Arslan

**Affiliations:** School of Engineering, The University of Edinburgh, Edinburgh EH9 3FF, UK; a.riaz-6@sms.ed.ac.uk (A.R.); isaied@exseed.ed.ac.uk (I.M.S.); z.shami@sms.ed.ac.uk (Z.S.)

**Keywords:** microwave sensing, planar monopole antenna, wideband, non-invasive sensors

## Abstract

Neurodegenerative diseases (NDs) can be life threatening and have chronic impacts on patients and society. Timely diagnosis and treatment are imperative to prevent deterioration. Conventional imaging modalities, such as Computed Tomography (CT), Magnetic Resonance Imaging (MRI), and Positron Emission Tomography (PET), are expensive and not readily accessible to patients. Microwave sensing and imaging (MSI) systems are promising tools for monitoring pathological changes, namely the lateral ventricle enlargement associated with ND, in a non-invasive and convenient way. This paper presents a dual-planar monopole antenna-based remote sensing system for ND monitoring. First, planar monopole antennas were designed using the simulation software CST Studio Suite. The antenna analysis was carried out regarding the reflection coefficient, gain, radiation pattern, time domain characterization, E-field distribution, and Specific Absorption Rate (SAR). The designed antennas were then integrated with a controlling circuit as a remote sensing system. The system was experimentally validated on brain phantoms using a vector network analyzer and a laptop. The collected reflection coefficient data were processed using a radar-based imaging algorithm to reconstruct images indicating brain abnormality in ND. The results suggest that the system could serve as a low-cost and efficient tool for long-term monitoring of ND, particularly in clinics and care home scenarios.

## 1. Introduction

Neurodegenerative disease (ND) is a progressive condition characterized by the gradual degeneration and death of nerve cells. It can manifest as dementia and brain strokes, posing life-threatening threats in acute situations and having chronic impacts on patients, families, and society in the long term. One of the most prevalent NDs is Alzheimer’s disease (AD), which is characterized by symptoms like memory loss, confusion with time and place, and problems in speaking and writing, which could severely disrupt daily life [1,2]. In the United States, it was estimated that 4.7 million individuals aged above 65 years have AD; with the ageing of the ‘baby boom’ generation, the number of people with AD is expected to increase dramatically in the next 40 years if no preventive measures are available [3]. This drastic increase in people suffering from the disease has become one of the great global healthcare challenges [1]. Typical pathological changes associated with ND include cerebral atrophy (the progressive shrinkage of the brain) [4,5] and lateral ventricle enlargement (LVE) [6,7]. These changes initiate in the early stages of the disease and deteriorate with time. Therefore, the extent of atrophy and LVE could serve as a marker for assessing disease progression. Imaging has played a key role in assessing and understanding these changes [1,8,9]. However, traditional modalities such as Computed Tomography (CT), Magnetic Resonance Imaging (MRI), and Positron Emission Tomography (PET) face limitations in accessibility in care homes or clinics for convenient long-term monitoring. Furthermore, CT scans involve ionizing radiation, and overexposure may lead to carcinogenicity. PET requires injecting radioactive liquid into the body [9,10]. The constraints of existing modalities necessitate the exploration of alternative approaches that are more convenient and non-invasive for ND monitoring.

### 1.1. Microwave Sensing and Imaging (MSI) Systems

Microwave sensing and imaging (MSI) for biomedical applications are emerging as non-intrusive and non-invasive means of examining functional and pathological tissue conditions [11,12,13]. MSI was introduced in 1973 for lung disease detection [14] and subsequently applied to different diseases [11,15], including breast cancers [16], lung cancers [17], and brain-related diseases such as brain injuries and stroke [11,15]. MSI techniques utilize the dielectric contrast of the tissues to reconstruct brain profiles and locate abnormalities. MSI systems typically consist of an antenna array, vector network analyzer or signal generator, and laptop, making it cost-effective and convenient for monitoring diseases. In addition, microwave systems could enable in-time diagnosis for pre-hospital scenarios and long-term monitoring for non-hospital scenarios, including care homes and community clinics. In the near future, MSI could also be integrated into on-site multimodal tools alongside other solutions such as ultrasound [18] and near-infrared spectroscopy [19] for hybrid sensing and imaging.

### 1.2. Wearable and Portable MSI Prototypes

MSI systems could be generally categorized into two types: wearable [20,21,22,23] and portable systems [24,25]. In [20], a flexible electromagnetic cap was proposed for brain stroke detection. The cap was integrated with a 24-element planar antenna array and subsequently connected to a multiport VNA. The wearable device was validated using brain phantoms with different stroke types. In [21], a wearable hat-shaped RF device consisting of six flexible antennas was proposed for detecting brain abnormalities associated with Alzheimer’s disease, namely brain atrophy and lateral ventricle enlargement. Validation was conducted using real lamb brains. In another study [22], a wearable device that integrated RF switching circuits was proposed. In the device, six one-pole-one-through (1P1T) solid-state switching circuits were utilized to manually switch among six antennas to connect to a two-port VNA. More recent work proposed a wearable glass embedded with four antennas for detecting neurodegenerative and cerebrovascular anomalies [23]. In [24], a microwave system consisting of 16 Vivaldi antennas was proposed for stroke detection. The Vivaldi antennas were evenly distributed on a circular platform and were connected to coaxial switching devices for sequential access to a two-port vNA. Realistic head phantoms were used for verification. Another portable microwave imaging system utilizing a rotating platform was reported in [25]. The system employed one antenna on the rotating platform to scan head models from 32 positions along a semicircular trace. As only one antenna was used, no switching circuits were required. Realistic human head phantoms were used to verify the system’s capabilities in detecting shallow and deep brain injuries. The above-mentioned systems were experimentally validated and successfully detected brain abnormalities. However, they could be further improved regarding user interface, hardware design and data collection methods.

This study aims to deploy portable microwave sensing and imaging devices for biomedical applications, specifically for neurodegenerative disease (ND) monitoring in care home and clinic scenarios. To achieve this, we propose a dual-planar monopole antenna-based remote sensing system. The remote sensing system utilized an antenna pair, an RF switching circuit, a Bluetooth module, an Arduino microcontroller, and a mobile app for controlling the antenna elements. The proposed system offers several advantages over existing microwave ND monitoring devices. Firstly, the portable system avoids direct contact with ND patients, particularly the elderly, enabling a more comfortable and less resistant measurement procedure. Secondly, automatic data collection is realized via the controlling circuit and the antenna pair. The designed controlling circuit and the antenna pair advance toward Internet of Things (IoT) solutions for healthcare in the elderly by circumventing the use of bulky switching networks and high-cost multi-port vector network analyzers (VNAs). As a proof of concept, we utilized a one-pole-four-throw switching circuit to switch between two antennas positioned at the side of brain phantoms. The antennas and controlling circuit were then placed on a portable platform in a sitting position for patients with ND. Scattering data were captured using the antenna pair and the remote-controlling circuit. The primary contributions of this work are outlined as follows:

- A customized planar monopole antenna was designed for ND monitoring. The antenna features a low profile and wideband characteristics, operating within the frequency range of 1 to 2.5 GHz to ensure sufficient penetration depth for detecting LVE in the brain’s lateral regions. The wideband capability was achieved through a tapered structure at the radiating patch and the feeding line. The antenna’s near-field performance, when placed close to brain phantoms, was evaluated in terms of time domain characterization, E-field distribution, and S-parameter. The Specific Absorption Rate (SAR) was also assessed to ensure the antenna adheres to safety limits.
- A remote-controlling circuit was designed for remotely accessing and commanding the antenna pairs. The circuit includes a microcontroller, one-pole-four-throw (1P4T), Bluetooth module and mobile app. The S-parameter data, specifically the reflection coefficients, were collected when the antenna pairs were positioned in close proximity to brain phantoms. Two data sets were collected: one was collected manually, and one was collected using the controlling circuit. The two sets of data were then compared and processed with a radar-based algorithm to verify the effectiveness of the proposed system.

This paper is organized as follows: Section 2 describes the planar monopole antenna’s design, simulation and fabrication. Section 3 evaluated antenna performance in terms of reflection coefficient, gain and radiation pattern. Section 4 presents the design and testing of the controlling circuit. Section 5 and Section 6 demonstrate the experimental setup using the antennas and the controlling circuit. The measured results are analyzed and processed with a radar-based algorithm to generate an energy map indicating the brain abnormalities, namely the lateral ventricular enlargement associated with ND. Finally, Section 7 concludes the paper and suggests future work.

## 2. Antenna Design, Simulation and Fabrication

The primary requirements for the antennas include a wideband for radar-based data processing, low profile to enhance the portability of MSI systems, and sufficient penetration depth to reach the brain lateral ventricular region [21,22,25]. To meet the requirements, a planar monopole antenna was designed to exhibit a wide operating band in the frequency range of 1 to 2.5 GHz. The monopole antenna consisted of a semi-tapered rectangular radiating patch and a coplanar waveguide (CPW) feeding line. The key empirical formulas for designing the antennas are presented below [26]. The width and length of the radiating patch were evaluated using Equations (1) and (2)

(1) $$W=\frac{c}{f_{r}\sqrt{2(\varepsilon_{r}+1)}}$$

where *W* refers to the rectangular patch width, *c* refers to the speed of light, $f_{r}$ refers to the resonant frequency of the antenna, and $\varepsilon_{r}$ refers to the relative permittivity of the antenna substrate.

(2) $$L=\frac{c}{2f_{r\sqrt{\varepsilon_{\mathrm{eff}}}}}-0.824h\left(\frac{\left(\varepsilon_{\mathrm{eff}}0.3\right)\left(\frac{W}{h}+0.264\right)}{\left(\varepsilon_{\mathrm{eff}}-0.258\right)\left(\frac{W}{h}+0.8\right)}\right)$$

where *L* refers to the length of the rectangular patch, *c* refers to the speed of light, $\varepsilon_{\mathrm{eff}}$ refers to the effective permittivity of the substrate, and *h* and *W* are the thickness and width of the substrate.

The effective permittivity of the substrate could be evaluated as:

(3) $$\varepsilon_{\mathrm{eff}}=\frac{\varepsilon_{r}+1}{2}+\frac{\varepsilon_{r}-1}{2\;\cdot \;\sqrt{1+12\;\cdot \;\frac{h}{W}}}$$

where $\varepsilon_{\mathrm{eff}}$ refers to the effective permittivity of the substrate, $\varepsilon_{r}$ refers to the relative permittivity of the substrate, and *h* and *W* refer to the thickness and width of the substrate, respectively.

The CPW feeding line impedance was 50 Ohm, the dimensions of which were evaluated using the simulation tool AppCad.

**Figure 1:**
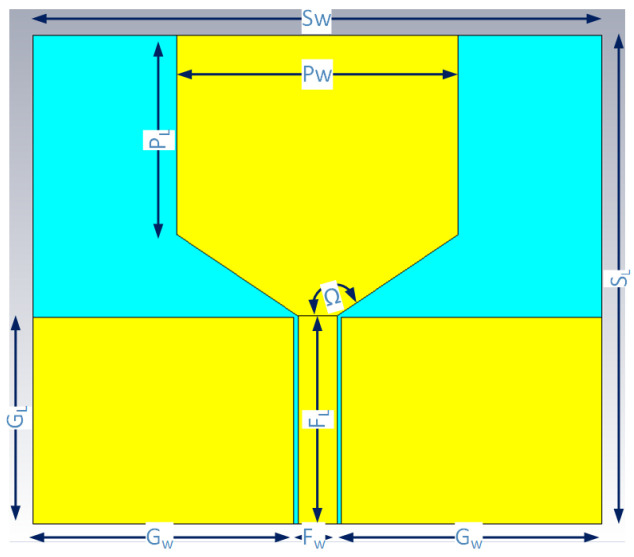
Geometry of the proposed monopole antenna. The antenna was fabricated on an ISO400 FR-4 substrate with a thickness of 1.55 mm, a dielectric constant of 3.9 and a loss tangent of 0.022. The radiating patch and feeding line were etched on a 35 μm copper (golden color) on the top of the substrate (green color).

**Figure 2:**
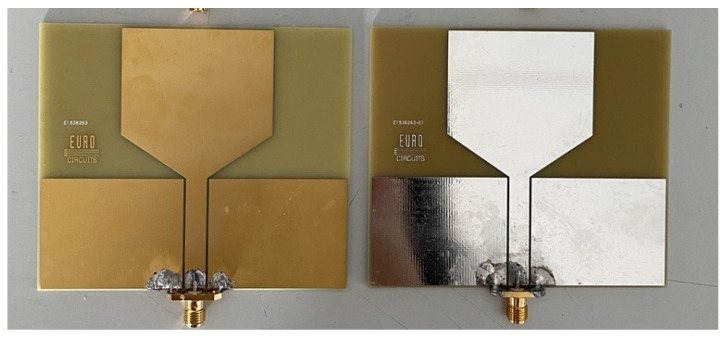
Fabricated monopole antenna prototype.

The antenna radiating patch dimension was calculated by referencing at a central resonant frequency $f_{r}$ of 2 GHz. The initially designed rectangular patch antenna is naturally narrowband. The bandwidth limitation was addressed by introducing a tapered structure from the feeding line to the radiating patch. This modification facilitated a smooth current flow from the feeding line to the radiating patch, ensuring an increase in bandwidth. For the feeding line, a coplanar waveguide (CPW) feeding line was adopted for its low radiation loss, superior signal integrity, and ease of fabrication. The optimization of the feeding line width and radiating patch dimensions was conducted using the simulation software CST Studio Suite 2019. The geometry of the proposed monopole antenna is plotted in Figure 1, and the detailed dimension parameters are tabulated in Table 1. The antenna was fabricated on an ISO400 FR-4 substrate with a thickness of 1.55 mm, a dielectric constant of 3.9 and a loss tangent of 0.022. The radiating patch and feeding line were etched on a 35 μm copper on the top of the substrate. The end of the feeding line was soldered using a SubMiniature version A (SMA) coaxial connector. The fabricated prototypes of the monopole antennas are shown in Figure 2.

**Figure 3:**
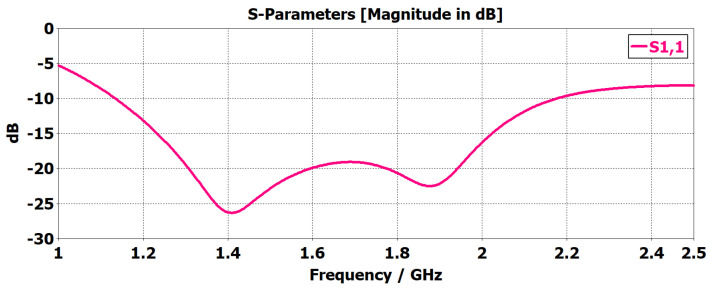
Simuated free space $S_{11}$ of the monopole antenna. The simulated free space $S_{11}$ indicated an operating frequency ranging from 1.2 to 2.2 GHz with a bandwidth of 1.0 GHz.

**Figure 4:**
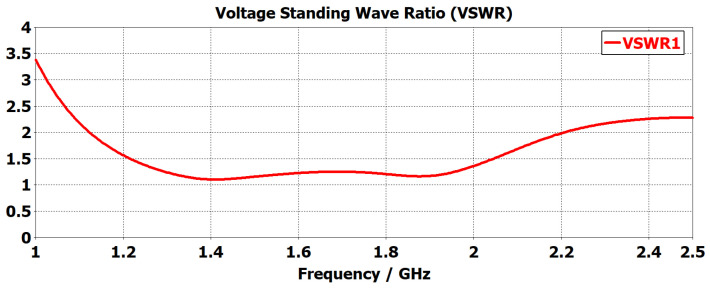
Simulated Voltage Standing Wave Ratio (VSWR) of the monopole antenna. The VSWR is below 2 within the frequency range.

**Figure 5:**
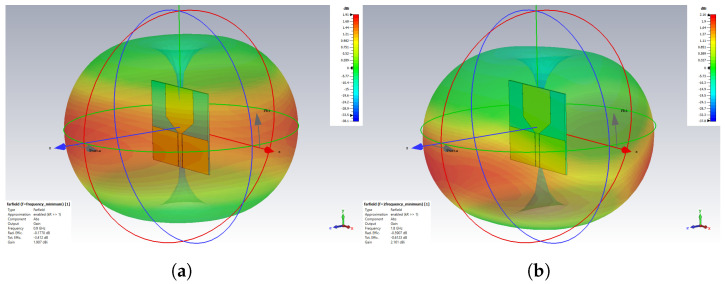
Simulated free space radiation pattern of the monopole antenna at (**a**) 0.9 GHz and (**b**) 1.8 GHz.

**Figure 6:**
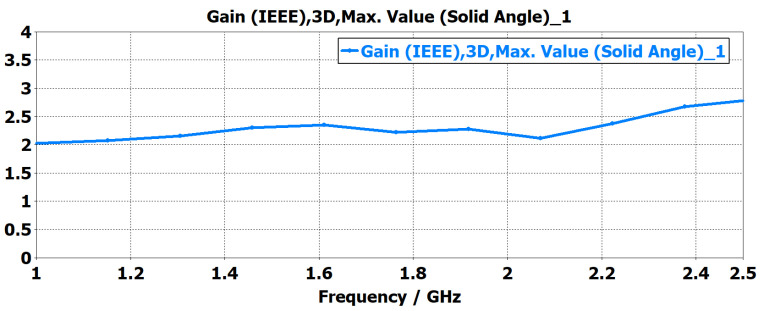
Simuated gain of the monopole antenna. The gain retains stable performance within the operating frequency range.

**Figure 7:**
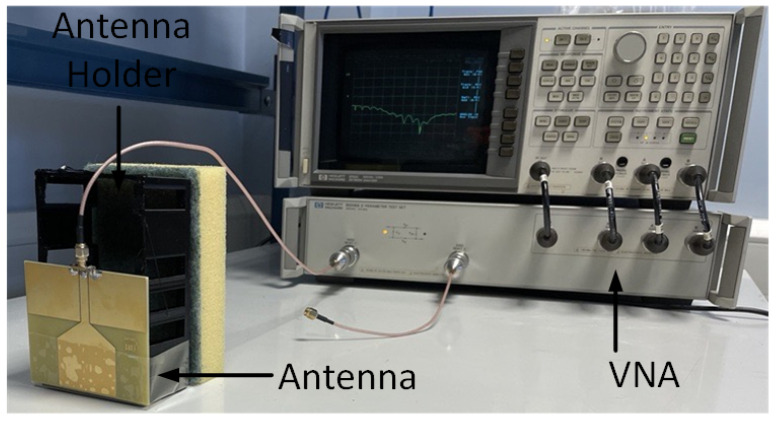
Reflection coefficient measurement setup using an HP8753C vector network analyzer (VNA). A plastic holder was placed at the back of the antenna to support the antenna. To reduce potential pick-up unwanted backlobe noise, an absorbing material is placed at the back.

**Figure 8:**
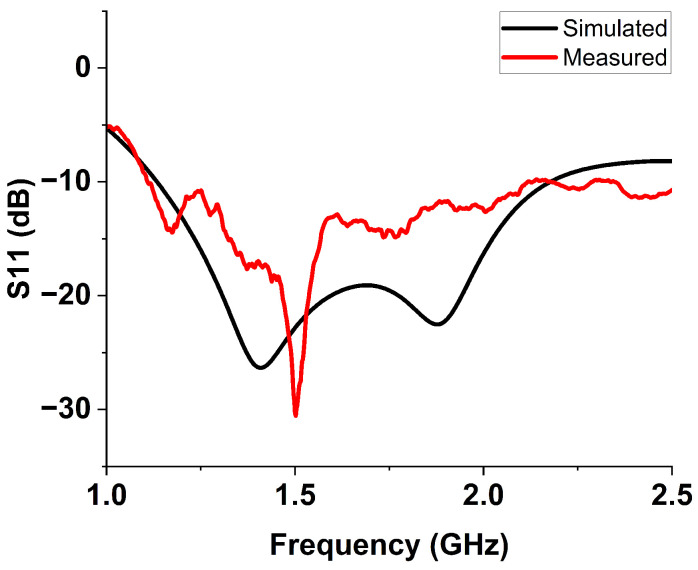
Simuated and measured free space $S_{11}$ of the monopole antenna. The measured $S_{11}$ showed an operating frequency from 1.1 to 2.5 GHz with 1.4 GHz bandwidth. The measured free space $S_{11}$ result showed good agreement with the simulated $S_{11}$ result in the operating band. The slight shift in resonant frequency might be due to fabrication and soldering tolerance.

The proposed monopole antenna was simulated and evaluated regarding reflection coefficient $S_{11}$, Voltage Standing Wave Ratio (VSWR), radiation pattern and gain in free space. The simulated free space $S_{11}$ indicated an operating frequency ranging from 1.2 to 2.2 GHz, with a bandwidth of 1.0 GHz, as shown in Figure 3. The Voltage Standing Wave Ratio (VSWR) is below 2 at this frequency range, as shown in Figure 4. The radiation pattern of the antenna indicated a bidirectional radiating direction toward the front and back of the radiating patch as indicated in Figure 5. The simulated gain shows stability across the operating frequency with slight variation from 2.03 to 2.78 dBi from 1 to 2.5 GHz, as plotted in Figure 6. The stability in gain across the frequency range is advantageous for radar-based imaging. The free space $S_{11}$ was measured using a vector network analyzer, as shown in Figure 7. The measured $S_{11}$ showed an operating frequency from 1.1 to 2.5 GHz with 1.4 GHz bandwidth. The measured free space $S_{11}$ result showed good agreement with the simulated $S_{11}$ result in an operating band with a slight shift in resonant frequency, as indicated in Figure 8. This might be due to fabrication and soldering tolerance.

## 3. Near-Field Analysis

For microwave applications, the field in which the antenna operates could be determined as shown in Equation (4) [27]. Let the antenna operating frequency be 2 GHz, the effective permittivity of the head model be 40, $\lambda_{m}$ refers to the wavelength in the head model at 2 GHz, the antenna largest dimension D be around 80 mm, and the calculated radiating near field region is estimated to be within 55 cm. In wearable and portable microwave systems, the antenna is placed in close proximity to the head. Given that the average male head circumference is around 56–58 cm, with a length of approximately 18–25 cm, it can be inferred that the monopole antenna operates in the near-field region.

(4) $$R=\frac{2D^{2}}{\lambda_{m}}$$

where *D* refers to the largest dimension of the antenna, and $\lambda_{m}$ refers to the EM wavelength in the medium.

**Figure 9:**
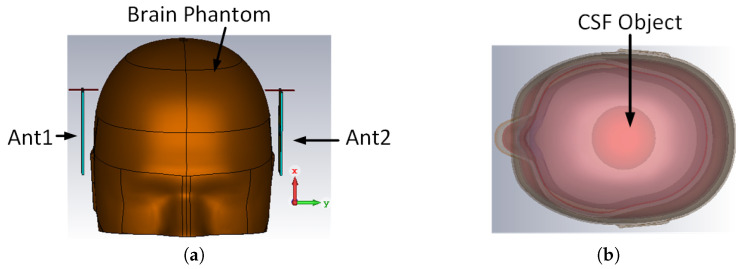
Realistic brain phantom used in simulation (**a**) front view of brain phantom, with two antennas placed at the side, and (**b**) top view, CSF object placed in the ventricular region to represent VLE.

Realistic brain phantoms were used to mimic the structure and dielectric properties of human brains [21]. The brain phantom consisted of the skin, skull, cerebral spinal fluid (CSF), gray matter, and white matter, as shown in Figure 9b. Lateral ventricle enlargement (LVE) was emulated by placing a 56 mL CSF object in the ventricular region. This emulation represented a mild stage of ND progression [6,22]. Simulations were conducted on healthy brain phantoms and brain phantoms with LVE, with an antenna pair placed at the side of the brain phantoms, as shown in Figure 9a.

### 3.1. Time Domain Characterization

**Figure 10:**
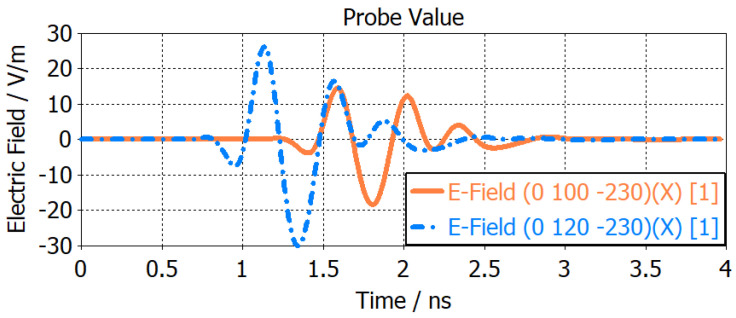
Simuated time-domain E-field strength inside the brain phantom at 20 mm and 40 mm from the antenna.

Time domain characterization of the antenna was simulated by placing E-field probes at a distance of 20 mm and 40 mm inside the brain phantom. The E-field pulse used was a default Gaussian pulse, the shape of which was determined by indicating the frequency range in CST. As shown in Figure 10, a delay and reduction in E-field strength was observed as the EM wave propagates into the brain phantom due to the impact of the tissue layers. The time domain E-field results validated the efficacy of the antenna in near-field performance.

### 3.2. E-Field Distribution and Scattering Behaviour

**Figure 11:**
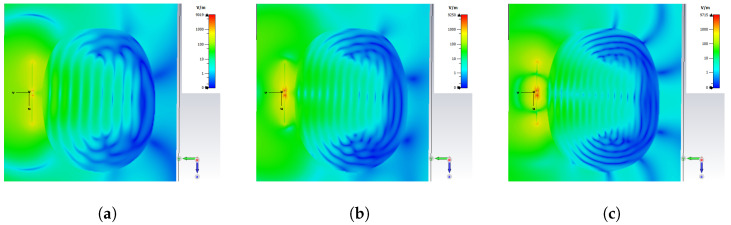
Simulated E-field distribution in the brain phantom at the frequency of (**a**) 1.5 GHz, (**b**) 2 GHz, and (**c**) 2.5 GHz.

The penetration depth into the brain phantom can be visualized by the antenna E-field distribution. The penetration depth of the monopole antenna was evaluated at the frequencies of 1.5, 2, and 2.5 GHz, as shown in Figure 11. At these frequencies, the wave generated from the antenna propagated through skin, skull gray matter, and white matter and reached through the lateral ventricular regions. The E-field results indicated that the the monopole antenna attained sufficient penetration depth for brain ventricular region sensing.

**Figure 12:**
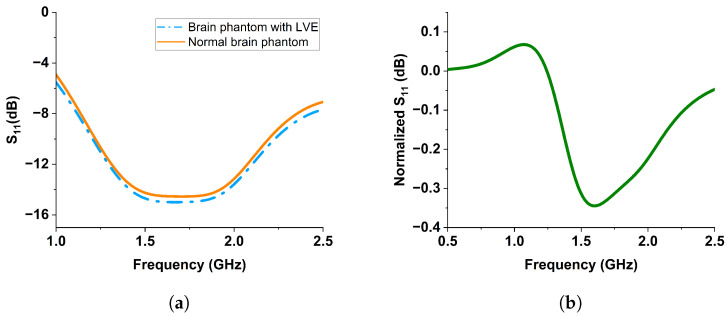
(**a**) The simulated reflection coefficients of the antenna when placed near the normal and abnormal brain phantoms, (**b**) by referencing the normal brain phantom, the normalized $S_{11}$ was obtained to represent the signal change caused by LVE.

The scattering behavior in the presence of the LVE was evaluated through reflection coefficient $S_{11}$. The simulated $S_{11}$ for both the normal brain phantom and the brain phantom with LVE is shown in Figure 12a. Within the frequency range of 1.5 to 2.0 GHz, a downward shift in $S_{11}$ could be observed with the presence of LVE. By referencing the normal brain phantom, the $S_{11}$ was normalized to represent the signal change caused by LVE, as plotted in Figure 12b.

### 3.3. Health and Safety Evaluation

Microwaves may raise safety concerns for humans depending on the strengths, operational frequencies, and exposure times of the field [28]. Within the frequency range of 300 GHz, the primary known adverse effect of EM fields (EMFs) on human tissue is heating due to the localized absorption close to the surface. The body is incapable of regulating its temperature above a specific exposure limit. The level of exposure is characterized by the Specific Absorption Rate (SAR), which measures the amount of EM radiation absorbed by human tissue. SAR can be evaluated using Equation (5):

(5) $$\mathrm{SAR}=\frac{\sigma \;\cdot \;E^{2}}{\rho }$$

where σ (unit: Siemens/meter) refers to the material’s electrical conductivity, *E* (unit: Volts/meter) refers to the E-field magnitude, and ρ (unit: kg/m^3^) refers to brain tissue mass density.

**Figure 13:**
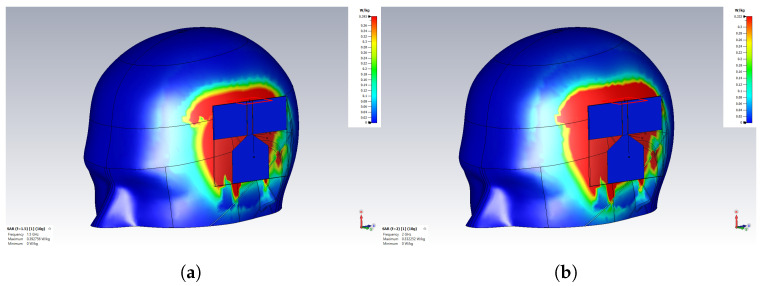
Simulated Specific Absorption Rate (SAR) on the brain phantom with an input power of 100 mW at the frequency of (**a**) 1.5 GHz and (**b**) 2 GHz.

The IEEE C95.1 standard [29] specifies the maximum local SAR as 2 W/Kg per 10 g of tissue on the head for the general public for frequencies between 100 KHz and 3 GHz. For the monopole antenna, the SAR was calculated by averaging over 10 g tissue in simulation, as shown in Figure 13. SAR was evaluated at an input power of 100 mW at the frequency of 1.5 GHz and 2 GHz, as plotted in Figure 13. The simulated SAR values with an input power of 100 mW and 500 mW are tabulated in Table 2. The table shows that the maximum SAR was 1.96 W/Kg with 500 mW input power. Benchmarking on the 2 W/Kg per 10 g of tissue SAR limit, all the simulated SAR values were within the safety limit. Hence, the antennas are safe for medical applications when operating below 500 mW.

## 4. Controlling Circuit Design

**Figure 14:**
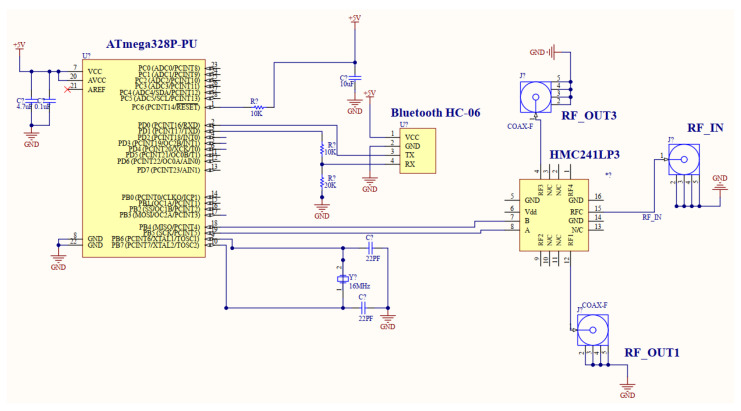
Schematic diagram of the controlling circuit: the microcontroller ATMEGA328P-PU is connected to the HMC241ALP3E 1P4T RF switch using two digital control lines. Additionally, it is connected to the Bluetooth HC-06 module using two communication lines.

To realize automatic control of the monopole antennas, a controlling circuit was developed utilising an RF switching circuit, a microcontroller, a Bluetooth module, and a mobile app. A microcontroller Atmega328P-PU was used to control an HMC241LP3 one-pole four-throw (1P4T) RF switch. The communication with the RF switch was established via a Bluetooth HC-06 module and a mobile app. The schematic of the controlling circuit is depicted in Figure 14, where the microcontroller ATMEGA328P-PU was connected to the HMC241ALP3E 1P4T RF switch using two digital control lines and to the Bluetooth HC-06 module using two communication lines. Specifically, pin 18(PB4), pin 19(PB5) of the microcontroller were connected to pin 7(B),8(A) of the HMC241LP3 1P4T switch, and pin 2(RXD) and pin 3(TXD) of the microcontroller were connected to pin 3(TX), 4(RX) of the Bluetooth HC-06 module. Pin 4 (RF3) and pin 12 (RF1) of the 1P4T were then connected to the two coaxial cables, and the cables were then connected to the antenna pairs, namely Ant1 and An2 at $RF_{OUT3}$ and $RF_{OUT1}$. The pin 15(RFC) of the 1P4T was connected to VNA through another coaxial cable at $RF_{IN}$. In the experiment, the controlling circuit was realized using commercial off-the-shelf (COTS) components. An Arduino board was adopted as the microcontroller for its compactness and ease of use. An HMC241ALP3E one-pole four-throw (1P4T) RF switch evaluation board from analog devices was selected as it aligned with the operating frequency of the antennas.

## 5. Experimental Validation

The antenna pair and the controlling circuit were integrated into a remote sensing system. The system was tested on fabricated brain phantoms representing normal and LVE conditions. The brain phantom fabrication and measurement setup are described below.

### 5.1. Brain Phantom

**Figure 15:**
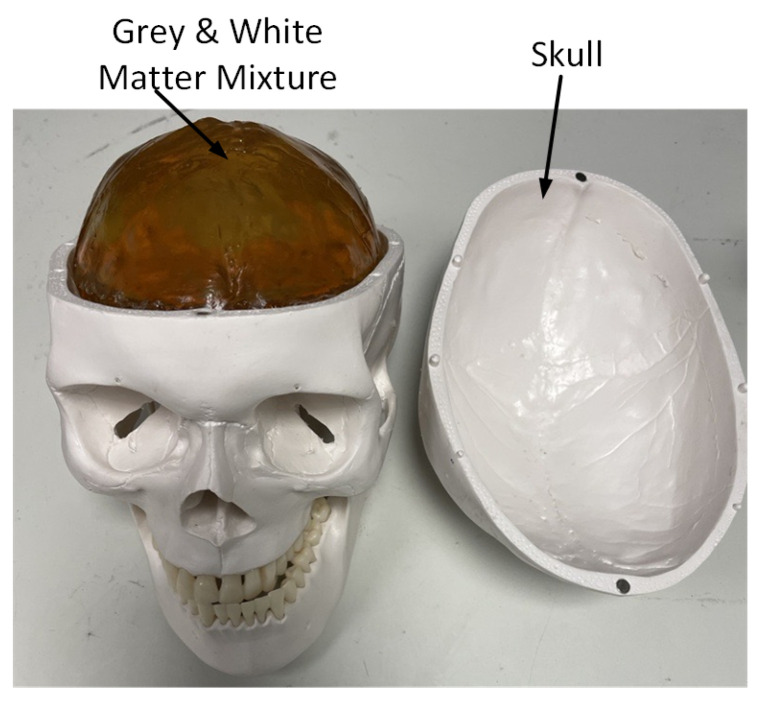
Overview of the fabricated brain phantom, a life-size skull was used to contain the gray and white matter mixture.

Brain phantoms were fabricated using the recipe outlined in Table 3 [22]. A homogeneous material was fabricated to emulate the average dielectric properties of a gray and white matter mixture. A life-size skull was used to contain the gray and white matter mixture. The superficial skin layer was omitted by assuming it has negligible effects on EM wave propagation in the head. LVE was emulated by carving the gray and white matter mixture and replacing the carved part with a 56 mL Cerebrospinal Fluid (CSF) object to match the brain phantom simulation settings. The CSF object was made from sodium chloride aqueous solutions [30] with a dielectric constant of 68 and a conductivity of 5 S/m. The fabricated brain phantom is illustrated in Figure 15 The dielectric properties of the brain phantom were validated using an Agilent high-temperature dielectric probe 85070E-0020.

### 5.2. Measurement Set-Up

**Figure 16:**
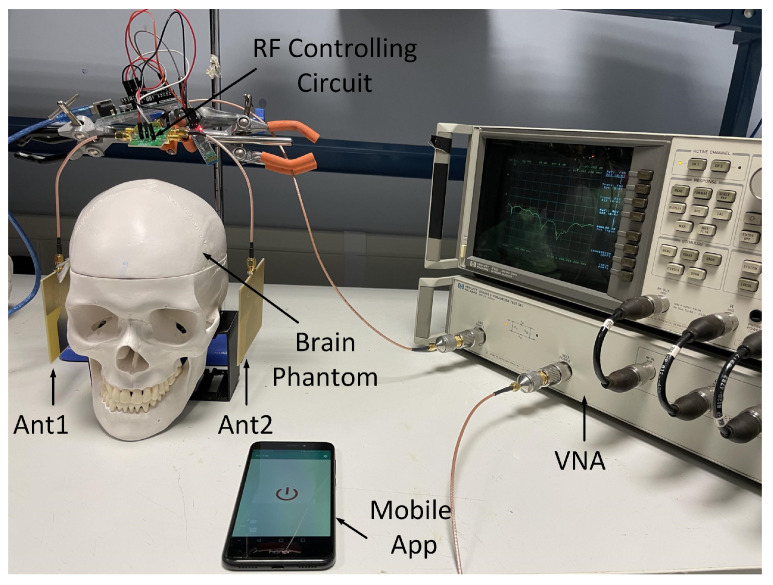
Measurement set up for capturing reflection coefficients using an HP8753C vector network analyzer (VNA), the controlling circuit and a mobile app. Ant1 and Ant2 are placed at the two sides of the brain phantom.

The measurement set-up is illustrated in Figure 16, where the antennas designed in the previous section were connected to the two RF paths of the RF controlling module, namely RF1 and RF3. The inputs of the RF control circuit are cascaded to the ports of an HP8753C VNA. The data from the VNA were collected with a laptop through a General Purpose Interface Bus (GPIB). For the 8753C VNA, 201 points were selected with an input power of 0 dBm and a frequency range of 0.3 to 3000 MHz. A full two-port calibration was performed before measurement. The RF controlling circuit and the antennas were mounted on a stand. The antenna pairs, namely Ant1 and Ant2, were initially positioned on the side of the brain phantoms and later re-positioned at the front and back of the brain phantoms. During measurement, EM absorbers were placed at the back of the antennas to prevent unwanted back lobe noise.

## 6. Results and Discussion

The effectiveness of the 1P4T switch was evaluated prior to the S-parameter measurement. Afterwards, the S-parameter data were subsequently collected using the set-up depicted in Figure 16. The first group of S-parameter data was collected manually without using the controlling circuit, and the second group of S-parameter data was collected utilizing the controlling circuit. For each group of S-parameter data, the reflection coefficients were collected from Ant1 and Ant2 both from normal brain phantoms and brain phantoms with LVE.

### 6.1. Switching Circuit Evaluation

To verify the performance of the RF switch, Return Loss (RL) and Insertion Loss (IL) were evaluated when the paths were in the ‘on’ state. Return loss refers to the power loss caused by the signal reflection of the device; insertion loss refers to the power loss at the insertion of the device. In the frequency range of 1 to 2.5 GHz, the measured return loss of both paths, RF1 and RF3, is well below −15 dB, and the measured insertion loss is above −2 dB. The measured average RL and IL of the 1P4T from 1 to 2.5 GHz is shown in Table 4. This confirmed the impedance matching of the 1P4T switching circuit in the required frequency range.

### 6.2. S-Parameter Results Discussion

**Figure 17:**
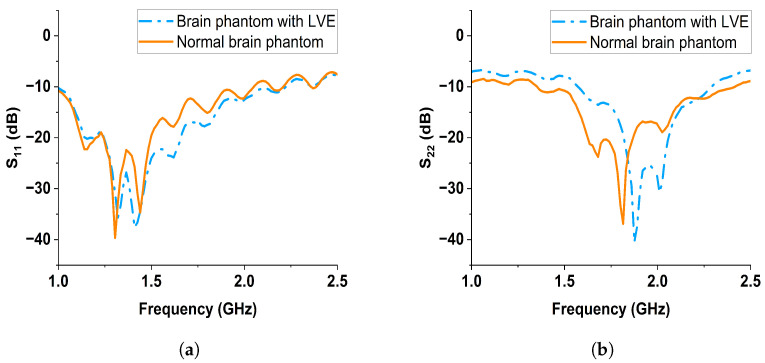
Reflection results collected manually (without the controlling circuit) of the normal brain phantom and brain phantom with LVE from (**a**) Ant1, (**b**) Ant2.

**Figure 18:**
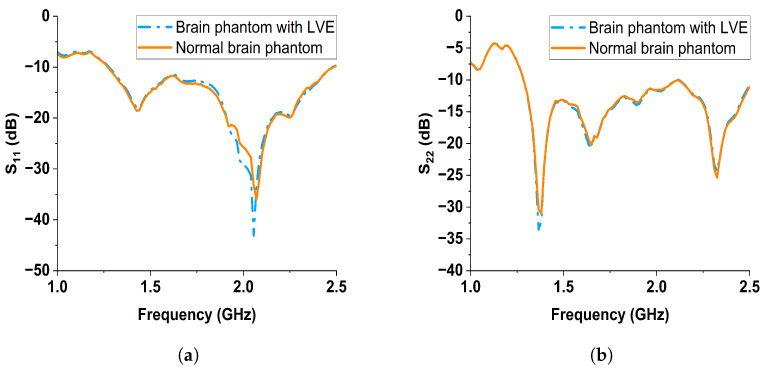
Reflection results collected with the controlling circuit of the normal brain phantom and brain phantom with LVE from (**a**) Ant1, (**b**), Ant2.

Two sets of reflection coefficient data were obtained: one collected manually and one using the controlling circuit. The reflection coefficients collected at the two sides of brain phantoms using Ant1 and Ant2 are denoted by $S_{11}$ and $S_{22}$, respectively. The data collected manually are plotted in Figure 17a,b; the data collected with the switching circuit are plotted in Figure 18a,b. From the manual results, both the reflection coefficients $S_{11}$ and $S_{22}$ from Ant1 and Ant2 showed noticeable changes between the normal brain phantom and brain phantom with LVE, especially at the frequency range of 1 to 2.2 GHz. When the controlling circuit is added, the reflection coefficient changes of Ant1 and Ant2 were less significant than the manual results. Nonetheless, the $S_{11}$ from Ant1 shows a downward shift from 1.8 to 2.1 GHz, while the $S_{22}$ from Ant2 shows a downward shift from 1.3 to 2.0 GHz. The changes in the reflection coefficient were attributed to the significant dielectric property change along the microwave propagation path.

**Figure 19:**
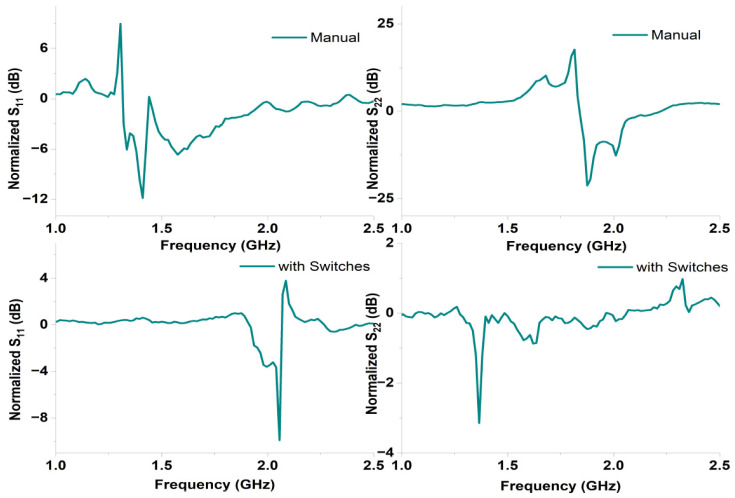
Normalized reflection coefficients $S_{11}$, $S_{22}$ of Ant1 and An2 collected manually and using the switches of the controlling circuit.

The reflection data were normalized by referencing the normal brain phantom to represent the signal change caused by LVE. The four sets of normalized reflection coefficient data collected from Ant1 and Ant2 are plotted in Figure 19 with the top two subfigures representing the manual results and the bottom two subfigures representing the results collected using the controlling circuit. All the four sets of normalized reflection coefficients exhibited noticeable changes when LVE was present. Comparing the manual results with those obtained using the switching circuit, the measured results showed a lower reflection coefficient magnitude. Despite the reduced magnitude, the measured reflection data indicated the feasibility of utilizing the controlling circuit for measurements. The performance variance between the antenna pair Ant1 and Ant2 might be due to the inconsistencies in antenna fabrication and SMA connector soldering.

### 6.3. Image Reconstruction

The collected reflection coefficient data were utilized to reconstruct images indicating LVE. The images were generated using Microwave Imaging via Space-Time (MIST) beamforming algorithm. The algorithm was initially proposed for breast cancer detection [31] and is adopted for brain abnormalities imaging. The normalized frequency domain data from Figure 19 were converted to the time domain using Inverse Fast Fourier Transform (IFFT) and time-aligned. The MIST algorithm was subsequently applied to the time domain data.

Firstly, the location of the antennas was represented in Cartesian coordinates as:

(6) $$L_{n}=\left[X_{n},Y_{n}\right]$$

where *n* represents the *n*th antenna.

The delay of each pixel to the antennas was then calculated as:

(7) $$\tau_{n}\left(\vec{r}\right)=\left(\frac{\sqrt{{\left(X_{n}-X_{0}\right)}^{2}+{\left(Y_{n}-Y_{0}\right)}^{2}}}{a}\right)N$$

where $X_{n}$ and $Y_{n}$ represent the coordinates of the nth *n*th antenna, $X_{i}$ and $Y_{i}$ represent the coordinates of each pixel, *a* the diameter and *N* the number of input data samples.

The delay values of the antennas to each pixel were put in a matrix and used as input to calculate the intensity values of the pixels:

(8) $$I\left[n\right]={\left|\sum_{i=1}^{N}A_{n}\left(\tau_{n}\left(\vec{r}\right)\right)\right|}^{2}$$

where *N* is the total amount of antennas and $A_{n}$ represents the intensity at location $X_{i}$ and $Y_{i}$.

**Figure 20:**
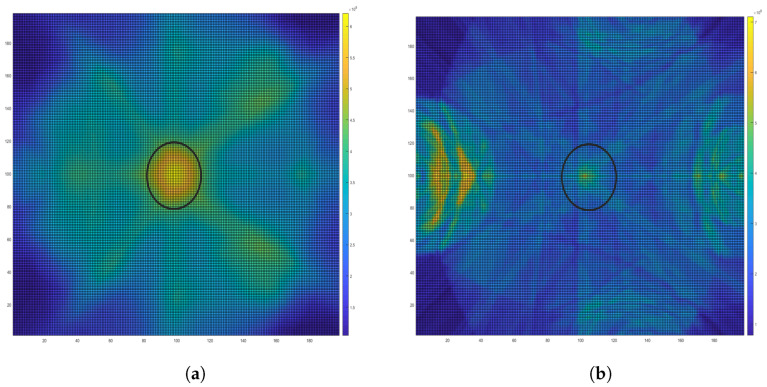
The reconstructed image indicates lateral ventricle enlargement, as shown in the circled area, using measured reflection results that were collected (**a**) manually and (**b**) with the switching circuit.

Applying the MIST algorithm created an energy map to indicate brain abnormality, namely the LVE region, as shown in Figure 20. Figure 20a was created from the data collected manually, and Figure 20b was created from data collected from the controlling circuit. Both figures successfully detected LVE. However, Figure 20b has a lower resolution attributed to the extra noise introduced by the controlling circuit.

The performance comparison of the proposed remote sensing system is compared to existing radar-based MSI systems, as tabulated in Table 5.

## 7. Conclusions & Future Work

This paper presented a dual-planar monopole antenna-based remote sensing system for microwave medical applications, specifically ND monitoring. Firstly, a customized, low-profile and wideband planar monopole antenna was designed, and the numerical analysis of the antenna was carried out in simulation software CST Studio Suite concerning gain and radiation pattern, time domain characterization, E-field distribution, and reflection coefficient. The Specific Absorption Rate (SAR) was also evaluated to ensure the antenna aligned with the safety limits. The designed antenna was integrated into a controlling circuit comprising a microcontroller, 1P4T RF switching circuit, Bluetooth module, and mobile app to enable automatic control. Experimental validation was conducted on normal brain phantoms and brain phantoms associated with ND. Reflection measurements were collected manually and using the controlling circuit from the brain phantoms. The collected data were processed using a radar-based imaging algorithm to indicate the brain abnormality, namely the lateral ventricular enlargement region. The reconstructed images demonstrated the system’s capability to detect brain abnormalities associated with ND. This suggests that the device could serve as a low-cost and efficient tool for long-term monitoring of patients with ND in clinics and care home scenarios. Future work aims to enhance the compactness of the controlling circuit by integrating the switching circuits, the microcontroller and the Bluetooth module into a single board. Additionally, the controlling circuit will be expanded to measure transmission and reflection coefficient data around brain phantoms. Methods for imaging algorithm optimization will also be explored to enhance the image quality.

## Figures and Tables

**Table 1:** Dimension parameters of the proposed antenna.

Dimension Parameters	Symbol	Value
Substrate Length	$S_{l}$	75.70 mm
Substrate Width	$S_{w}$	88.00 mm
Substrate Thickness	$S_{t}$	1.55 mm
Patch Length	$P_{l}$	30.89 mm
Patch Width	$P_{w}$	43.49 mm
Ground Length	$G_{l}$	32.00 mm
Ground Width	$G_{w}$	40.35 mm
Feedline Length	$F_{l}$	32.21 mm
Feedline Width	$F_{w}$	6.00 mm
Copper Thickness	$C_{t}$	0.035 mm
Tapered Structure Angle	Ω	146°

**Table 2:** Specific Absorption Rate (SAR) of monopole antenna (per 10 g).

SAR (W/Kg)	1.5 GHz	2 GHz
100 mW	0.39	0.33
500 mW	1.96	1.66

**Table 3:** Recipe for the brain phantom.

Material	Quantity (mL)
Water	700
Sugar	600
Gelatin	100

**Table 4:** Measured average RL and IL of the 1P4T from 1 to 2.5 GHz.

RF Path	Return Loss (dB)	Insertion Loss (dB)
RF1	−23.25	−0.39
RF3	−23.77	−0.76

**Table 5:** Comparison of wearable and portable MSI systems.

System	Platform	Antenna Type	Antenna Number	Frequency Band (GHz)	Targetting Disease
20	Wearable cap	Planar multi-slot	24	0.9–2.5	Stroke
22	Wearable hat	Planar monopole	6	0.6–0.8 & 1.8–2.09	ND
23	Wearable glass	Planar monopole	4	1.2–2.5	ND & Stroke
24	Circular platform	Vivaldi	16	1–4	Stroke
25	Rotatble platform	Slotted dipole	1	1.1–3.4	Brain injury
This Work	Portable platform	Planar monopole	2	1.2–2.2	ND

## Data Availability

Data available upon request.

## Abbreviations

The following abbreviations are used in this manuscript:

ND	Neurodegenerative
CT	Computed Tomography
MRI	Magnetic Resonance Imaging
PET	Positron Emission Tomography
MSI	Microwave Sensing and Imaging
MIST	Microwave Imaging via Space–Time
SMA	SubMiniature version A
LVE	Lateral Ventricular Enlargement
CSF	Cerebrospinal Fluid
CPW	Coplanar Waveguide
SAR	Specific Absorption Rate
VNA	Vector Network Analyzer
1P1T	One-Pole One-Throw
1P4T	One-Pole Four-Throw
RL	Return Loss
IL	Insertion Loss
